# Clustering protein sequences with a novel metric transformed from sequence similarity scores and sequence alignments with neural networks

**DOI:** 10.1186/1471-2105-6-242

**Published:** 2005-10-03

**Authors:** Qicheng Ma, Gung-Wei Chirn, Richard Cai, Joseph D Szustakowski, NR Nirmala

**Affiliations:** 1 Biomedical Computing, Genome and Proteome Sciences, Novartis Institutes for BioMedical Research, Inc. Cambridge, MA 02139 USA

## Abstract

**Background:**

The sequencing of the human genome has enabled us to access a comprehensive list of genes (both experimental and predicted) for further analysis. While a majority of the approximately 30000 known and predicted human coding genes are characterized and have been assigned at least one function, there remains a fair number of genes (about 12000) for which no annotation has been made. The recent sequencing of other genomes has provided us with a huge amount of auxiliary sequence data which could help in the characterization of the human genes. Clustering these sequences into families is one of the first steps to perform comparative studies across several genomes.

**Results:**

Here we report a novel clustering algorithm (CLUGEN) that has been used to cluster sequences of experimentally verified and predicted proteins from all sequenced genomes using a novel distance metric which is a neural network score between a pair of protein sequences. This distance metric is based on the pairwise sequence similarity score and the similarity between their domain structures. The distance metric is the probability that a pair of protein sequences are of the same Interpro family/domain, which facilitates the modelling of transitive homology closure to detect remote homologues. The hierarchical average clustering method is applied with the new distance metric.

**Conclusion:**

Benchmarking studies of our algorithm versus those reported in the literature shows that our algorithm provides clustering results with lower false positive and false negative rates. The clustering algorithm is applied to cluster several eukaryotic genomes and several dozens of prokaryotic genomes.

## Background

Clustering of protein sequences from different organisms has been used to identify orthologous and paralogous protein sequences, to find protein sequences unique to an organism, and to derive the phylogenetic profile for a cluster of protein sequences. These are some of the essential components of a comparative genomics study of protein sequences across several genomes.

**Figure 1:**
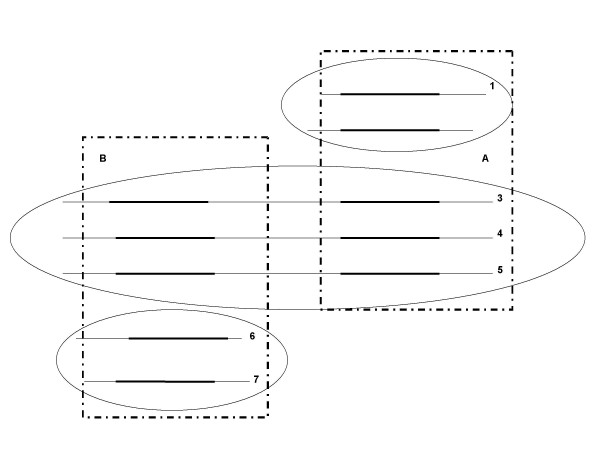
**The schematic view of family-based clustering**. Figure 1 illustrates a typical example of the clustering of three protein families denoted by the three oval outlines. Family I consists of protein sequences 1 and 2. Family II consists of protein sequences 3, 4, and 5. Family III consists of protein sequence 6 and 7. Domain A is common to families 1 and 2 while Domain B is common to families 2 and 3.

The methods of clustering protein sequences can be either domain-based or family-based. All the clustering methods start with an all-against-all pairwise protein sequence similarity search. The domain-based clustering methods organize the protein sequence universe into domain clusters where domains are the structural units of proteins, e.g., COG [1], ProDom [2], and Picasso [3] (Figure 1). A multidomain protein may belong to multiple domain clusters.

Clusters of Orthologous Groups (COGs) find triangles of mutually consistent genome-specific best hits from distant organisms without specifying a fixed similarity cut-off, thus accommodating both fast evolving and slow evolving genes. It then merges triangles which share a common edge. Each COG cluster is further analyzed manually to eliminate false positives caused by multidomain proteins so that each COG cluster represents a domain.

ProDom is based on the assumption that short protein sequences are single domain proteins. It first sorts all the protein sequences according to their lengths. It then undergoes a repetitive process: during each iteration, ProDom chooses as the query sequence the current shortest protein sequence or its internal repeat unit if it has internal repeats, searches the whole protein sequence set with PSI-BLAST [4], builds the sequence profile, and masks segments covered by the sequence profile for multidomain proteins or removes the single domain proteins completely covered by the sequence profile. The process iterates until there is no sequence left in the protein sequence set.

Picasso merges pairwise sequence alignments from the initial all-against-all pairwise sequence similarity searches into multiple sequence alignments of closer homologs, and later hierarchically merges these multiple sequence alignments into representative sequence profiles of remote homologs by profile-profile comparisons. The representative sequence profiles may contain sequences of different domain structures, but share at least one domain. Picasso then cuts domains within the representative sequence profiles into individual domain clusters based on the concept of overlapping maximal clusters proposed in SYSTERS [5]. Maximal clusters are clusters not fully contained in any other clusters. Two maximal clusters may have not only the overlapping set of neighbour members but also the unique set of neighbour members to these two maximal clusters. Thus these two overlapping maximal clusters must be of different domain structures sharing at least one domain which corresponds to the overlapping set of neighbour members. Then these two overlapping maximal clusters must undergo domain-cutting to be split into individual domains corresponding to closed neighbours, where no member has any neighbour outside of the cluster, from multiple alignments. However, since it is still a challenging problem to precisely pinpoint the structure domain border based on primary sequence information [6,7], the performance of the clustering algorithm will be determined by the accuracy of domain demarcations.

Family-based clustering methods group protein sequences into families, which contain a group of evolutionarily related proteins that share similar domain architecture (see Figure 1), e.g., CluSTr [8], SYSTERS, ProClust[9,10], PROTONET [11]/ProtoMap [12], and MCL[13]. CluSTr clusters protein sequence with the single linkage algorithm using the Z-score as the metric.

SYSTERS uses each protein sequence in the dataset as a seed sequence and applies the single linkage algorithm with a stringent threshold. Thus, each seed sequence has a cluster associated with it. It then merges all the clusters to maximal clusters. The maximal clusters could be either separate maximal clusters corresponding to single domain protein clusters or overlapping maximal clusters representing clusters having multiple domains, but sharing at least one domain.

ProClust uses a different metric to detect whether the aligned two proteins have similar domain structures. The metric value, which scales from 0 to 1, is the ratio of the raw score of the sequence alignments to the raw score of one of those two sequences aligned to itself. Thus the metric value between two sequences is directional. It assumes that the metric is symmetric if two aligned sequences have similar domain structure and non-symmetric otherwise. It then represents each sequence as a vertex and represents the metric value above the threshold as a directional edge in a directed graph. Each strongly connected component corresponds to a cluster [9]. It was later improved by building Profile-HMMs for all clusters having more than 20 sequences and merging two clusters A and B into a cluster corresponding to a SCOP superfamily if the average E-value from searching all the sequences in the cluster A against the profile-HMM of the cluster B is below the threshold[10].

PROTONET applies the hierarchical clustering of protein sequences based on the their pairwise similarity E-values, but adopts different rules for merging clusters: arithmetic mean, geometric mean, and harmonic mean. However, different families may have different levels of sequence conservation. It is not appropriate to choose one E-value threshold. And at the level of higher E-value, it may merge two clusters of different domain structures, but sharing one domain. However, different families may have different levels of sequence conservation. It is not appropriate to choose one E-value threshold. And at the level of higher E-value, it may merge two clusters of different domain structures, but sharing one domain.

Transitive homology detection methods have been proposed in the Intermediate Sequence Search, ISS [14,15], and [16]. It works by searching the query sequence against the database with a conservative threshold to find the closely homologous sequences and using these homologous sequences as seeds to search the database to find remotely homologous sequences with a less conservative threshold. The method has been shown to be close to the profile based methods and better than a direct pairwise homology search [17]. But, it is a challenge to quantify the indirect, transitive homology as opposed to using the E-value for quantifying direct pairwise sequence homology.

The Markov cluster (MCL) [13] algorithm has been successfully applied to clustering protein sequences. MCL represents protein sequences as nodes on a graph where similar proteins are connected by edges weighted according to their BLASP E-Value. The MCL algorithm works through a series of iterative random walks across the graph and inflations of the edge weights that gradually strengthens the connections between very similar nodes and weakens the connections between less similar nodes. MCL makes no explicit use of protein domain architecture but does leverage transitive homologies in the random walk phase of the algorithm.

Compared to the hierarchical clustering family based clustering method, e.g., PROTONET [11], our method can take advantage of the transitive homologue closure by the third intermediate sequence to detect remote homologues at the superfamily level. Compared to single linkage based methods, e.g., CluSTr [8], our method avoids the problem of merging two unrelated multi-domain cluster sharing a common domain. Compared to the iterative clustering method, e.g., SYSTERS [5], our method generates clusters where each sequence belongs to only one cluster.

## Results and discussion

### Benchmarking

**Figure 2:**
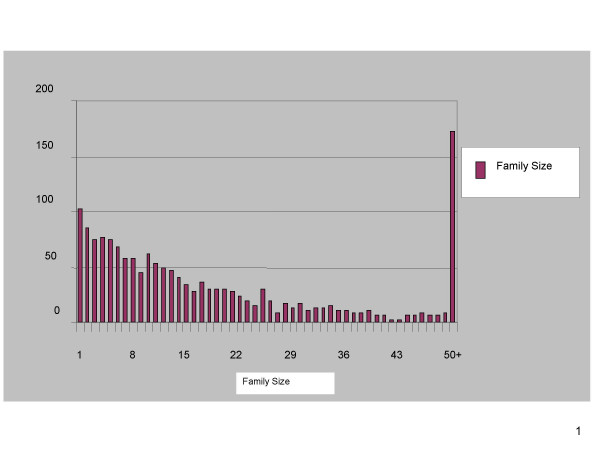
**shows the distribution of InterPro family size**. Figure 2 shows the distribution of the InterPro families used in the benchmarking dataset based upon the number of members in each family. There are 102 singleton InterPro families, and the largest InterPro family in the benchmarking dataset is Rhodopsin-like GPCR superfamily which has 1058 protein sequences in the benchmarking dataset.

In order to test the performance of CLUGEN, we selected all Swissprot [18] sequences with an InterPro [19] annotation, which resulted in 41480 sequences from 1598 InterPro families. The criteria we used to select sequences are that more than one member database from the InterPro annotation have the same superfamily or domain assignment and that the aligned region of the Swissprot sequence with respect to either Profile or hidden Markov model is longer than 30 amino acids. The benchmarking dataset is available on request. Figure 2 shows the InterPro superfamily/domain size distribution in the benchmarking dataset. There are 102 singleton families, that is families that consist of only one sequence. The largest family is IPR000276, the Rhodopsin-like G-Protein Coupled Receptor (GPCR) family which comprised of 1058 protein sequences.

### Performance measure

**Figure 3:**
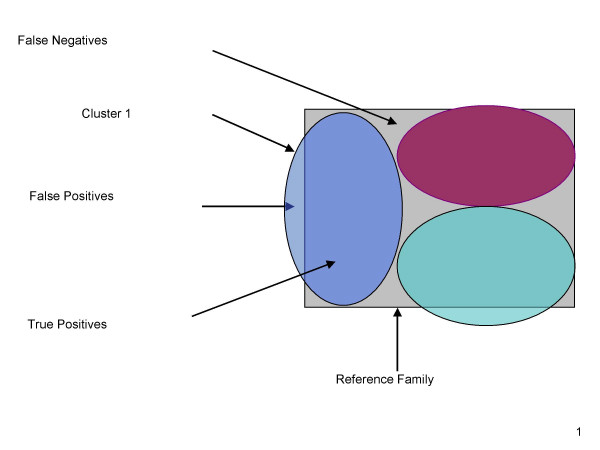
**Definition of various clustering parameters**. Figure 3 illustrates the mapping of three generated clusters denoted by oval outlines differentiated by different colors into a InterPro family denoted by a rectangle. The cluster can be mapped to an InterPro family only if more than 50% cluster members belong to that InterPro family; and is declared as a orphan cluster otherwise. Protein sequences outside the rectangle are false positives. Protein sequences within both the oval outline and the rectangle are true positives. Protein sequences wholly within the grey rectangle are false negatives.

We measure CLUGEN's performance by sensitivity, specificity, and goodness. A protein sequence is a false positive (*FP*) if it is misclassified to a certain InterPro superfamily/domain and a true positive (*TP*) otherwise. A protein sequence in a certain InterPro superfamily/domain is a false negative (*FN*) if it is not classified to that InterPro superfamily/domain (Figure 3). Let *N*_*fp *_and *N*_*tp *_denote the number of false positives and the number of true positives with respect to a cluster. Let *N *_*fn *_denote the number of false negatives with respect to a InterPro superfamily/domain.

Specificity: The ***specificity ***of a cluster is defined as *N*_*tp *_/ (*N*_*tp *_+ *N*_*fp*_).

Sensitivity: The ***sensitivity ***of an InterPro superfamily/domain is defined as *N*_*tp *_/ (*N*_*tp *_+ *N*_*fn *_).

Goodness: The ***goodness ***of an InterPro superfamily/domain is a measure of how well a cluster corresponds to the mapped InterPro superfamily/domain and is defined below (Equation 1) where *N *denotes the number of generated clusters associated with that InterPro superfamily/domain. The Area Under the ROC Curve (AUC) has been shown to be a better evaluation measure than accuracy within the context of binary classification, where the negative dataset is clearly defined. However, we cluster protein sequences into 1598 interpro families simultaneously. As a result, using the AUC as a measure of performance is not the appropriate metric here. Instead, we adopt as the "goodness " the set relative measure as defined in [12]. In order to decrease the goodness value when a large number of clusters is associated with an InterPro superfamily/domain, a penalty of (N-1) is applied in the numerator of the equation.

Ideally *specificity, sensitivity*, and *goodness *should be 100%.

Equation 1:

Goodness=∑i=1NNtpi−N+1∑i=1NNtpi+∑i=1NNfpi+Nfn
 MathType@MTEF@5@5@+=feaafeart1ev1aaatCvAUfKttLearuWrP9MDH5MBPbIqV92AaeXatLxBI9gBaebbnrfifHhDYfgasaacH8akY=wiFfYdH8Gipec8Eeeu0xXdbba9frFj0=OqFfea0dXdd9vqai=hGuQ8kuc9pgc9s8qqaq=dirpe0xb9q8qiLsFr0=vr0=vr0dc8meaabaqaciaacaGaaeqabaqabeGadaaakeaacqWGhbWrcqWGVbWBcqWGVbWBcqWGKbazcqWGUbGBcqWGLbqzcqWGZbWCcqWGZbWCcqGH9aqpdaWcaaqaamaaqahabaGaemOta40ccqWG0baDcqWGWbaCdaWgaaqaaiabdMgaPbqabaGccqGHsislcqWGobGtcqGHRaWkcqaIXaqmaSqaaiabdMgaPjabg2da9iabigdaXaqaaiabd6eaobqdcqGHris5aaGcbaWaaabCaeaacqWGobGtliabdsha0jabdchaWnaaBaaabaGaemyAaKgabeaakiabgUcaRmaaqahabaGaemOta40ccqWGMbGzcqWGWbaCdaWgaaqaaiabdMgaPbqabaGccqGHRaWkcqWGobGtliabdAgaMjabd6gaUbqaaiabdMgaPjabg2da9iabigdaXaqaaiabd6eaobqdcqGHris5aaWcbaGaemyAaKMaeyypa0JaeGymaedabaGaemOta4eaniabggHiLdaaaaaa@66DA@

### Overall performance

We evaluated CLUGEN at several threshold values. Table 1 lists shows the specificity, sensitivity, and goodness as well as the number of generated clusters and the number of orphan clusters as a function of the different threshold values respectively. A cluster can be mapped to an InterPro family only if more than 50% of the cluster members belong to that InterPro family; and is declared as an orphan cluster otherwise. At one extreme of the spectrum, each cluster is a singleton cluster consisting of only one protein sequence. Thus both specificity and sensitivity are 100%. But the goodness value is very low, the reciprocal of the size of the family. Clustering based on more stringent threshold values, e.g. 0.5, generates a larger number of smaller clusters causing a smaller number of false positives, also resulting in a low goodness value. As the threshold values become less stringent, small clusters merge at different levels into larger clusters, therefore a smaller number of larger clusters are generated. At the threshold of 0.2, there are 1706 clusters resulting in a specificity of 97.1%, sensitivity 98.6%, goodness value of 78.2%, and the number of orphan clusters is 201. As can be seen from table 1, the threshold value is a compromise of sensitivity, specificity, goodness and the number of orphan clusters. Ideally, we would like the clustering results to produce as many clusters as there should be and as few orphan clusters as possible.

**Table 1:** Specificity, sensitivity, goodness, cluster number, and orphan cluster values at different cutoff values on the benchmarking dataset.

cutoff	specificity	sensitivity	goodness	cluster number	Number of orphan clusters
0.20	97.11%	98.60%	78.20%	1706	201
0.22	97.37%	98.70%	78.00%	1742	180
0.25	97.61%	98.70%	77.60%	1786	157
0.29	97.85%	98.70%	76.90%	1837	133
0.33	98.06%	98.90%	76.30%	1896	107
0.40	98.43%	99.00%	75.00%	1972	79
0.50	98.70%	99.10%	72.60%	2073	59

**Figure 4:**
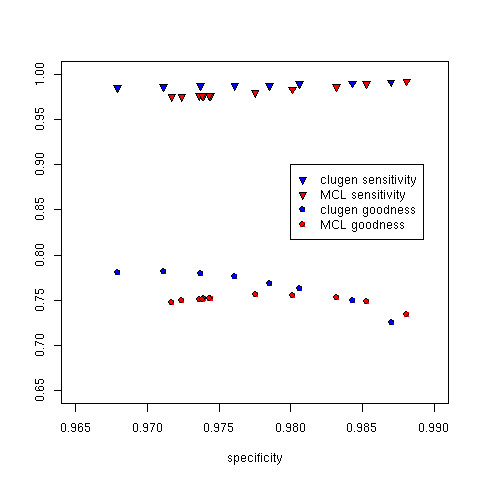
**Specificity, sensitivity, and goodness on the benchmarking dataset**. Sensitivity and specificity for CLUGEN and MCL at various specificities. At higher specificities, the sensitivity of both methods increases, whereas the goodness of both methods decreases. This is expected because higher specificities are achieved via stricter parameter thresholds that more clusters overall and fewer large clusters. Performance for both methods is comparable in this range with CLUGEN performing better at lower specificities and MCL performing better at higher specificities.

**Figure 5:**
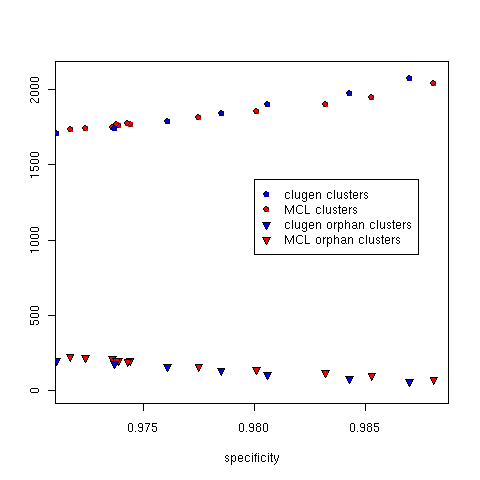
**The number of generated clusters and orphan clusters on the benchmarking dataset**. Total clusters and orphan clusters for clugen and MCL at various specificities. With stricter parameter thresholds, overall specificity and the total number of clusters increases for both methods. The larger number of small clusters at higher specificities leads to a reduction in the number of orphan clusters in both methods.

For a basis of comparison we also applied the MCL [13] algorithm to the same test dataset with various inflation values. Results are depicted in Figures 4 and 5. At higher specificities, the sensitivity of both methods increases. This is expected because higher specificities are achieved via stricter thresholds that result in more clusters overall and fewer large clusters. In the extreme case one could place each test sequence in its own cluster of size 1 and achieve 100% sensitivity and 100% specificity but with a low goodness score. This trade-off between sensitivity, specificity, and goodness is clearly evident in Figure 4; as specificity increases, sensitivity increases whereas goodness decreases.

In Figure 5 we see additional tradeoffs between specificity and overall performance. As specificity increases the number of orphan clusters decreases. This improvement in performance comes with an increase in the total number of clusters. Once again the extreme case of one sequence per cluster guarantees no orphan clusters at the cost of many non-informative clusters. Ideally one wishes to strike a balance reducing the number of orphan clusters while not drastically increasing the total number of clusters.

The overall performances of MCL and CLUGEN are fairly similar, with CLUGEN demonstrating a clear advantage at specificities below 0.98. CLUGEN's sensitivity and goodness are better at specificities below 0.98, whereas MCL's goodness is slightly better at specificities greater than 0.98. The number of total clusters and orphan clusters generated by both methods are comparable at specificities below 0.98. CLUGEN tends toward fewer orphan clusters at the cost of more total clusters at higher specificities.

### Analysis of some CLUGEN generated clusters

In this section, we will give examples of some successfully generated clusters with one-to-one correspondence to specific InterPro families, some clusters which have false positives, and some which have false negatives.

As previously outlined, 41480 sequences with Interpro superfamily annotation (1598 clusters) were clustered using our algorithm. This results in a total of 1972 clusters. Overall, there are 1199 clusters that have been correctly mapped with one-to-one correspondence to 1199 out of 1598 InterPro superfamilies /domains. There are 79 orphan clusters. Some correctly clustered large protein superfamily/domain examples are: 507 Cytochrome P450 proteins are correctly clustered into family IPR001128 without false positives and false negatives; 398 large chain ribulose bisphosphate carboxylase proteins are correctly clustered into family IPR000685 without false positives and false negatives; 290 short-chain dehydrogenase/reductase SDR proteins are clustered into family IPR002198 without false positives and false negatives. Table 2 shows top 50 InterPro superfamily/domains that have been mapped to clusters with one-to-one correspondence.

**Table 2:** Top 50 InterPro superfamily/domains that have been mapped to clusters with one-to-one correspondence

InterPro family/Domain ID	Type	Number of proteins in the benchmark dataset	Description
IPR001128	Family	507	Cytochrome P450
IPR000685	Family	398	Ribulose bisphosphate carboxylase, large chain
IPR002198	Family	290	Short-chain dehydrogenase/reductase SDR
IPR004000	Family	255	Actin/actin-like
IPR002423	Family	226	Chaperonin Cpn60/TCP-1
IPR001023	Family	221	Heat shock protein Hsp70
IPR002085	Family	181	Zinc-containing alcohol dehydrogenase superfamily
IPR000173	Family	177	Glyceraldehyde 3-phosphate dehydrogenase
IPR001175	Family	169	Neurotransmitter-gated ion-channel
IPR000910	Family	169	HMG1/2 (high mobility group) box
IPR001353	Family	147	20S proteasome, A and B subunits
IPR000894	Family	141	Ribulose bisphosphate carboxylase, small chain
IPR000298	Family	135	Cytochrome c oxidase, subunit III
IPR001019	Family	135	Guanine nucleotide binding protein (G-protein), alpha subunit
IPR000568	Family	134	H+-transporting two-sector ATPase, A subunit
IPR001400	Family	133	Somatotropin hormone
IPR000883	Family	131	Cytochrome c oxidase, subunit I
IPR001364	Family	131	Hemagglutinin, HA1/HA2 chain
IPR00970	Family	130	Secreted growth factor Wnt protein
IPR001664	Family	127	Intermediate filament protein
IPR000847	Domain	127	Bacterial regulatory protein, LysR
IPR001659	Family	124	Phycobilisome protein
IPR001694	Family	123	Respiratory-chain NADH dehydrogenase, subunit 1
IPR001811	Family	119	Small chemokine, interleukin-8 like
IPR000215	Family	118	Proteinase inhibititor I4, serpin
IPR001926	Family	114	Pyridoxal-5'-phosphate-dependent enzyme, beta subunit
IPR000515	Family	113	Binding-protein-dependent transport systems inner membrane component
IPR001424	Family	112	Copper/Zinc superoxide dismutase
IPR001804	Family	111	Isocitrate/isopropylmalate dehydrogenase
IPR001691	Domain	109	Glutamine synthetase, catalytic domain
IPR000934	Domain	105	Metallophosphoesterase
IPR001189	Family	105	Manganese and iron superoxide dismutase
IPR001041	Domain	105	Ferredoxin
IPR001099	Family	104	Naringenin-chalcone synthase
IPR001450	Domain	102	4Fe-4S ferredoxin, iron-sulfur binding domain
IPR001427	Family	102	Pancreatic ribonuclease
IPR000484	Family	100	Photosynthetic reaction centre protein
IPR000954	Family	98	Aminotransferase class-III
IPR001576	Family	93	Phosphoglycerate kinase
IPR000230	Family	93	Ribosomal protein S12, bacterial and chloroplast form
IPR002068	Domain	91	Heat shock protein Hsp20
IPR001750	Domain	90	NADH/Ubiquinone/plastoquinone (complex I)
IPR000836	Domain	90	Phosphoribosyltransferase
IPR001993	Family	90	Mitochondrial substrate carrier
IPR001236	Family	85	Lactate/malate dehydrogenase
IPR002210	Family	83	Papillomavirus major capsid L1 (late) protein
IPR001395	Family	81	Aldo/keto reductase
IPR000943	Family	80	Sigma-70 factor
IPR002226	Family	80	Catalase
IPR001766	Domain	80	Fork head transcription factor

We also conducted a detailed analysis of clusters that had false negatives/false positives in order to understand the areas in which the clustering algorithm could be further improved. The following is a description of errors encountered in clustering algorithms with specific reference to the data from our method.

#### Errors from low-complexity and coiled-coil regions

The first type of error is due to the presence of low complexity sequences with repetitive sequence patterns or sequences with coiled-coil structures, since we mask the low complexity regions and coiled-coil regions before the all-against-all pairwise similarity searches. As an example, the InterPro family IPR000533, Tropomyosins, which regulate muscle contraction, are alpha-helical proteins that form a coiled-coil. There are 25 tropomyosin sequences in the benchmarking dataset, among which 24 tropomyosin sequences are false negative sequences and appear in the following cluster along with members of IPR002699 ATP synthase subunit D.

#### Errors from short sequences or from an abundance of certain amino acid type in the sequences

Short sequences with less than 70 amino acids could also cause false positives in the clustering results. Cluster 1259 which is mapped to InterPro family 003019, the metallothionein superfamily, consists of 125 short protein sequences with 68 amino acids on average in length among which 34 false positive protein sequences are from InterPro family IPR001762, Disintegrin, and 34 false positive protein sequences are from InterPro family IPR000877, Bowman-Birk serine protease inhibitor. The reason these families cluster together is that metallothioneins are small proteins with high content of cysteine residues, while disintegrins and Bowman-Birk serine protease inhibitors are also short cysteine-rich protein sequences. This suggests that a more stringent threshold should be applied to cluster short protein sequences which are rich in a particular amino acid.

#### Similar domain structures in different superfamilies

Sequences that belong to different superfamilies but share similar domain structures may also cluster incorrectly in some cases. For example, 1039 out of 1058 sequences correctly cluster into the IPR000276 Rhodopsin-like G-protein coupled receptors family. But 17 out of 19 sequences from the IPR000276 Rhodopsin-like G-protein coupled receptors which have Cysteine-rich N-terminal regions are mistakenly clustered into the InterPro 000372 which is annotated as a cysteine-rich flanking region N-terminal. Similarly, members of the IPR001878 CCHC Zinc finger domain have been incorrectly clustered into the cluster 1008 which is mapped to the InterPro family 000981 Neurohypophysical hormone because they share the two cysteine residues and other surrounding weak motifs.

## Conclusion

This paper describes a novel method for the clustering of protein sequences based on a new metric derived from prediction using neural networks and further utilizing the metric to model the transitive sequence homologue to detect the remote homologue. Good performance with respect to the InterPro protein sequence database has been achieved on the benchmarking dataset.

Clustering of sequences has many applications in target discovery and gene functionalization. One can identify *in silico*, antimicrobial drug targets by examining clusters without any eukaryotic member sequence in it. These proteins could be potentially selective targets for infectious diseases due to the absence of any appreciable homolog in the human proteome. Such computationally derived targets need to be further validated using experimental data derived from gene expression profiling and proteomics experiments [20]. Another application is to predict the function for prokaryotic proteins of unknown function by phylogenetic profiling [21], where the phylogenetic profile for a cluster is a vector of binary values, with 1 meaning the presence of a genome in that cluster and 0 otherwise. The assumption here is that genes with the same phylogenetic profile could have the same function.

## Method

### Feature extraction

After we mask the low complexity regions and the coiled-coil regions and carry out the all-against-all pairwise sequence similarity searches, we extract four sets of features to represent the homology between any given pair of sequences. The first two sets of input features detect the homology of two aligned sequences, the last two sets of input features test whether two aligned sequences have similar domain structures. We use neural networks to map input features to a new metric, a probability value which scales from 0 to 1 and is interpreted as the likelihood that two sequences are of the same homologous superfamily.

The first input feature is the log scale of the pairwise E-value.

The raw score, from which the E-value of the two aligned sequences is derived, is calculated by summing up the log score of each position in the alignment, which assumes that each position is independent of the other. However, in practice, it has been shown that two consecutive positions in the alignments are quite correlated [22]. To model the correlation between two consecutive positions in the alignment, we adopt the concept of the 2-gram encoding method [23]. Ideally, hydrophobic regions of one sequence should align with the hydrophobic regions of the other sequence, and hydrophilic regions should align with each other as well. Each position in the alignment could fall into one of four categories: residue identity denoted by *A*_1_, hydrophobic similarity denoted by *A*_2_, hydrophilic similarity denoted by *A*_3_, and everything else denoted by *A*_4_. Let *Len*_*a *_denote the total length of the alignment and *Occur*_*i*,*j *_denote the number of occurrences of *i *and *j*, where *i *is immediately followed by *j*, with *i *and *j *denoting any one of *A*_1_, *A*_2_, *A*_3_, or *A*_4 _respectively. Let *freq*_*i,j *_denote frequency of *i,j*, which is equal to *Occur*_*i,j*_/(*Len*_*a*_-1). Thus the second set of input features consists of *freq*_*i,j *_values of the alignment positions, which consists of 16 input feature values for a pair of aligned sequences since each of the two consecutive positions could be one of four possible values.

**Figure 6:**
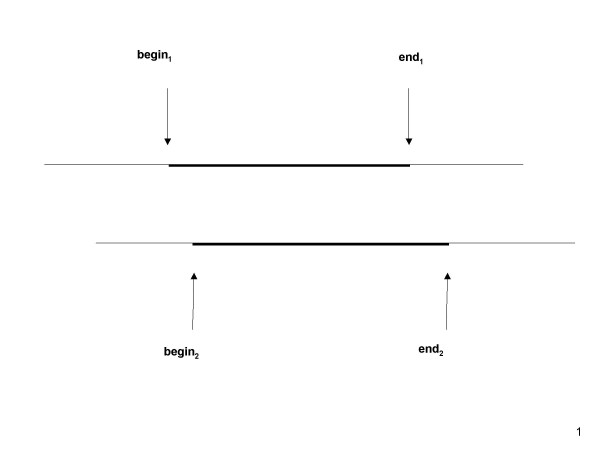
**A schematic view of a pairwise alignment**. Figure 6 shows a pairwise alignment between two aligned sequences. The aligned regions of the two sequences are highlighted. Their boundaries are pinpointed by the arrows.

Each of the two aligned sequences is separated into three segments, the unaligned N-terminal region, the aligned region, and the unaligned C-terminal region, by the beginning position of the alignment, denoted by *begin*_*i*_, and the end position of the alignment, denoted by *end*_*i *_with respect to *sequence*_*i *_(Figure 6). Let *Len*_*i *_denote the length of *sequence*_*i*_. Then lengths of three segments of *sequence*_*i *_are *begin*_*i *_-1, *end*_*i *_- *begin*_*i *_+1, and *Len*_*i *_- *end*_*i*_, respectively. If we normalize the length of each of three segments within an aligned *sequence*_*i *_by dividing the length of each segment by *Len*_*i*_, we get a vector of three values, *Seg*_*i*1 _= (*begin*_*i*_-1)/*Len*_*i*_, *Seg*_*i*2 _= (*end*_*i *_- *begin*_*i *_+1)/*Len*_*i*_, and *Seg*_*i*3 _= (*Len*_*i *_- *end*_*i*_)/*Len*_*i*_. Intuitively, if the two aligned sequences have similar domain structures, the alignment will divide the two aligned *sequences i and j *in a similar proportion, and the linear correlation coefficient, *LCC*_1 _defined by Equation 2, between these two vectors tend to be close to 1. So the third set of input features include *LCC*_1_, *Seg*_*i*1_, *Seg*_*i*2_, *Seg*_*i*3_, *Seg*_*j*1_, *Seg*_*j*2_, and *Seg*_*j*3_.

Equation 2:

LCC1=13∑k=1N(Segik−13)(Segjk−13)13∑k=1N(Segik−13)213∑k=1N(Segjk−13)2
 MathType@MTEF@5@5@+=feaafeart1ev1aaatCvAUfKttLearuWrP9MDH5MBPbIqV92AaeXatLxBI9gBaebbnrfifHhDYfgasaacH8akY=wiFfYdH8Gipec8Eeeu0xXdbba9frFj0=OqFfea0dXdd9vqai=hGuQ8kuc9pgc9s8qqaq=dirpe0xb9q8qiLsFr0=vr0=vr0dc8meaabaqaciaacaGaaeqabaqabeGadaaakeaacqWGmbatcqWGdbWqcqWGdbWqdaWgaaWcbaGaeGymaedabeaakiabg2da9maalaaabaWaaSaaaeaacqaIXaqmaeaacqaIZaWmaaWaaabCaeaacqGGOaakcqWGtbWucqWGLbqzcqWGNbWzdaWgaaWcbaGaemyAaKMaem4AaSgabeaakiabgkHiTmaalaaabaGaeGymaedabaGaeG4mamdaaiabcMcaPiabcIcaOiabdofatjabdwgaLjabdEgaNnaaBaaaleaacqWGQbGAcqWGRbWAaeqaaOGaeyOeI0YaaSaaaeaacqaIXaqmaeaacqaIZaWmaaGaeiykaKcaleaacqWGRbWAcqGH9aqpcqaIXaqmaeaacqWGobGta0GaeyyeIuoaaOqaamaakaaabaWaaSaaaeaacqaIXaqmaeaacqaIZaWmaaWaaabCaeaacqGGOaakcqWGtbWucqWGLbqzcqWGNbWzdaWgaaWcbaGaemyAaKMaem4AaSgabeaakiabgkHiTmaalaaabaGaeGymaedabaGaeG4mamdaaiabcMcaPmaaCaaaleqabaGaeGOmaidaaaqaaiabdUgaRjabg2da9iabigdaXaqaaiabd6eaobqdcqGHris5aaWcbeaakmaakaaabaWaaSaaaeaacqaIXaqmaeaacqaIZaWmaaWaaabCaeaacqGGOaakcqWGtbWucqWGLbqzcqWGNbWzdaWgaaWcbaGaemOAaOMaem4AaSgabeaakiabgkHiTmaalaaabaGaeGymaedabaGaeG4mamdaaiabcMcaPmaaCaaaleqabaGaeGOmaidaaaqaaiabdUgaRjabg2da9iabigdaXaqaaiabd6eaobqdcqGHris5aaWcbeaaaaaaaa@7C1C@

The fourth and final input feature is to measure the overlap between two neighbor sets of aligned sequences, where the neighbor set, *Set*_*i *_of *sequence*_*i*_, is defined as the set of protein sequences that *sequence*_*i *_hits when *sequence*_*i *_is used as the query sequence. One straightforward method to measure the overlap is to use the ratio of the cardinality of the intersection of two neighbor sets to the cardinality of the union of two neighbor sets.

Here we propose another method to measure the overlap. Specifically, if we represent the neighbor set of *sequence*_*i *_as *Vector*_*i*_, the value of the *k*^*th *^element of *Vector*_*i *_is the log E-value, *Log*_*ik *_between *sequence*_*i *_and its *k*^*th *^neighbor in *Set*_*i*_. However, *Vector*_*i *_and *Vector*_*j *_for two aligned sequences, *sequence*_*i *_and *sequence*_*j*_, may be of different dimensions since the cardinalities of *Set*_*i *_and *Set*_*j *_may be different. We make these two vectors have the same dimension by adding the log E-value threshold to *Vector*_*i *_whenever the *sequence*_*i *_has no corresponding neighbor in the neighbor set, *Set*_*j *_of the other aligned *sequence*_*j*. _Thus the last input feature is the linear correlation coefficient *LCC*_2 _between *Vector*_*i *_and *Vector*_*j *_defined by Equation 3. Intuitively, the more similar the domain structure two aligned sequences have, the more similar neighbor sets they will have, and the closer to 1 the linear correlation coefficient will be.

Equation 3:

LCC2=1N∑k=1N(Logik−Logi)(Logjk−Logj)1N∑k=1N(Logik−Logi)21N∑k=1N(Logjk−Logj)2
 MathType@MTEF@5@5@+=feaafeart1ev1aaatCvAUfKttLearuWrP9MDH5MBPbIqV92AaeXatLxBI9gBaebbnrfifHhDYfgasaacH8akY=wiFfYdH8Gipec8Eeeu0xXdbba9frFj0=OqFfea0dXdd9vqai=hGuQ8kuc9pgc9s8qqaq=dirpe0xb9q8qiLsFr0=vr0=vr0dc8meaabaqaciaacaGaaeqabaqabeGadaaakeaacqWGmbatcqWGdbWqcqWGdbWqdaWgaaWcbaGaeGOmaidabeaakiabg2da9maalaaabaWaaSaaaeaacqaIXaqmaeaacqWGobGtaaWaaabCaeaacqGGOaakcqWGmbatcqWGVbWBcqWGNbWzdaWgaaWcbaGaemyAaKMaem4AaSgabeaakiabgkHiTiabdYeamjabd+gaVjabdEgaNnaaBaaaleaacqWGPbqAaeqaaOGaeiykaKIaeiikaGIaemitaWKaem4Ba8Maem4zaC2aaSbaaSqaaiabdQgaQjabdUgaRbqabaGccqGHsislcqWGmbatcqWGVbWBcqWGNbWzdaWgaaWcbaGaemOAaOgabeaakiabcMcaPaWcbaGaem4AaSMaeyypa0JaeGymaedabaGaemOta4eaniabggHiLdaakeaadaGcaaqaamaalaaabaGaeGymaedabaGaemOta4eaamaaqahabaGaeiikaGIaemitaWKaem4Ba8Maem4zaC2aaSbaaSqaaiabdMgaPjabdUgaRbqabaGccqGHsislcqWGmbatcqWGVbWBcqWGNbWzdaWgaaWcbaGaemyAaKgabeaakiabcMcaPmaaCaaaleqabaGaeGOmaidaaaqaaiabdUgaRjabg2da9iabigdaXaqaaiabd6eaobqdcqGHris5aaWcbeaakmaakaaabaWaaSaaaeaacqaIXaqmaeaacqWGobGtaaWaaabCaeaacqGGOaakcqWGmbatcqWGVbWBcqWGNbWzdaWgaaWcbaGaemOAaOMaem4AaSgabeaakiabgkHiTiabdYeamjabd+gaVjabdEgaNnaaBaaaleaacqWGQbGAaeqaaOGaeiykaKYaaWbaaSqabeaacqaIYaGmaaaabaGaem4AaSMaeyypa0JaeGymaedabaGaemOta4eaniabggHiLdaaleqaaaaaaaa@8ABD@

where *N *is the dimension of *Vector*_*i *_and *Vector*_*j *_and *Log*_*i *_and *Log*_*j *_are the mean values and are defined by Logi=1N∑k=1NLogik
 MathType@MTEF@5@5@+=feaafeart1ev1aaatCvAUfKttLearuWrP9MDH5MBPbIqV92AaeXatLxBI9gBaebbnrfifHhDYfgasaacH8akY=wiFfYdH8Gipec8Eeeu0xXdbba9frFj0=OqFfea0dXdd9vqai=hGuQ8kuc9pgc9s8qqaq=dirpe0xb9q8qiLsFr0=vr0=vr0dc8meaabaqaciaacaGaaeqabaqabeGadaaakeaacqWGmbatcqWGVbWBcqWGNbWzdaWgaaWcbaGaemyAaKgabeaakiabg2da9maalaaabaGaeGymaedabaGaemOta4eaamaaqahabaGaemitaWKaem4Ba8Maem4zaC2aaSbaaSqaaiabdMgaPjabdUgaRbqabaaabaGaem4AaSMaeyypa0JaeGymaedabaGaemOta4eaniabggHiLdaaaa@42BC@, and Logj=1N∑k=1NLogjk
 MathType@MTEF@5@5@+=feaafeart1ev1aaatCvAUfKttLearuWrP9MDH5MBPbIqV92AaeXatLxBI9gBaebbnrfifHhDYfgasaacH8akY=wiFfYdH8Gipec8Eeeu0xXdbba9frFj0=OqFfea0dXdd9vqai=hGuQ8kuc9pgc9s8qqaq=dirpe0xb9q8qiLsFr0=vr0=vr0dc8meaabaqaciaacaGaaeqabaqabeGadaaakeaacqWGmbatcqWGVbWBcqWGNbWzdaWgaaWcbaGaemOAaOgabeaakiabg2da9maalaaabaGaeGymaedabaGaemOta4eaamaaqahabaGaemitaWKaem4Ba8Maem4zaC2aaSbaaSqaaiabdQgaQjabdUgaRbqabaaabaGaem4AaSMaeyypa0JaeGymaedabaGaemOta4eaniabggHiLdaaaa@42C0@.

To summarize, the first input feature is the log scale of the pairwise E-value. The second input feature consists of 16 feature values, which are frequency values of the alignment positions and the third input feature includes 7 values, which relate to the details of the alignments. And finally, the fourth input feature includes 1 feature value which measures the overlap of the neighbour sets to make a total of 25 input features which will be used to train the neural network as described below.

### Neural networks

After we represent the sequence homology between a pair of sequences by the set of 25 input features, we train the neural network using the training data. Each homologous pair of sequences is labeled as 1 if they belong to the same InterPro superfamily or the same domain if they are single domain proteins, and 0 otherwise. We selected as large a number of sequences as possible to train the neural networks to avoid overfitting the data. In all, we selected 27844 homologous sequence pairs as the positive training set and 29999 non-homologous sequence pairs as the negative training set.

**Figure 7:**
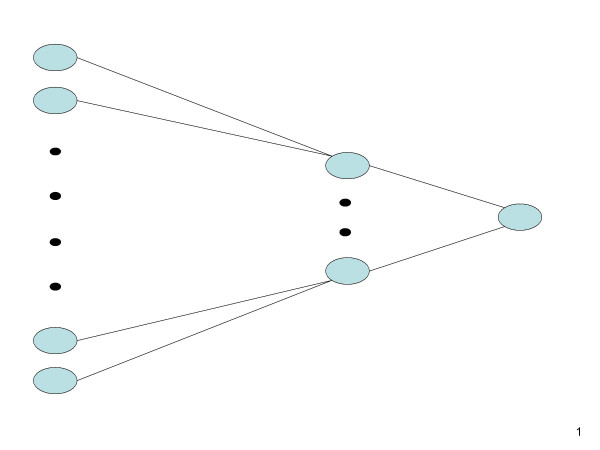
**The architecture of the neural network**. Figure 7 demonstrates the architecture of the neural network. The neural network is actually fully connected, but not shown in the figure for simplicity, and has three layers. The first layer is the input layer consisting of 25 input features. The hidden layer in the middle has 4 nodes. The output layer has one output node.

The neural network we use is a fully connected feed-forward back propagation neural network and has one hidden layer with sigmoid activation functions (see Figure 7). The output layer of the neural network has one output unit. The output value is bounded between 0 and 1. The network is trained with the scaled conjugate gradient algorithm [24] implemented in MATLAB [25].

Given the large amount of training data relative to the number of the weights in the neural network, the neural network is unlikely to overfit. It may however underfit if there are not a sufficient number of weights. If the training data are smaller relative to the number of weights in the neural network, measures should be taken to avoid the overfitting problem and the cross-validation method should be used to choose the best model. Clearly in this study, such is not the case.

We used a split-sample approach in which the validation set is not used during training, but is used to select the best model. After the neural network is trained, it is validated on the validation dataset containing 250597 homologous pairs of sequences and 30000 non-homologous pairs. Different numbers of hidden layer nodes have been tested. The ultimate goal is not to determine if any two proteins sequences belong to the same Interpro family, but to cluster all sequences in Interpro families as accurately as possible. We selected the model with the smallest number of weights and smallest error on the validation set. Thus, we chose 4 hidden layer nodes such that the neural network has the least number of hidden units and the best performance on the validation dataset with a specificity of 94.18% and a sensitivity of 91.81%.

### Modeling the transitive homology

**Figure 8:**
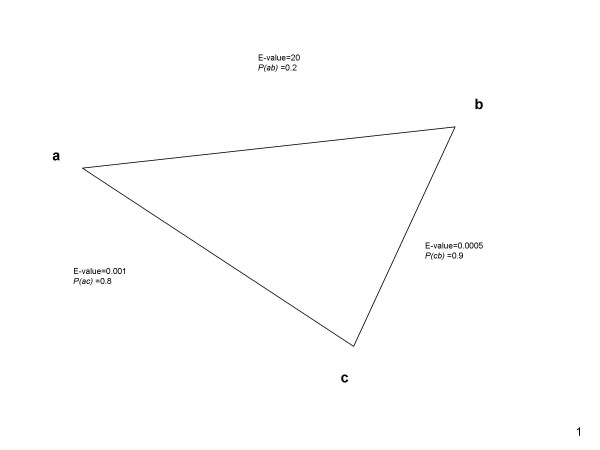
**Figure 8 illustrates the transitive homology between sequence a and sequence b through the third sequence c**. The homology between sequence *a *and sequence *b *can be detected with *P(a,b) *= 0.72 by the transitive sequence homology.

The neural network is then used to calculate the metric value for any pair of protein sequences that hit to each other below the E-value that was used as a cutoff. The metric value, *P(A,B)*, for protein sequences *A *and *B *is interpreted as the likelihood that these two protein sequences belong to the same InterPro superfamily or have the same single domain. The value *P(A,B) *is replaced by *P(A,C)P(C,B) *if there exists a sequence *C *such that *P(A,C)P(C,B) *is larger than the current value of *P(A,B)*. This transformation takes advantage of the transitive homology of sequences *A *and *B *through the intermediate sequence *C*, assuming that protein sequences *A *and *C *and protein sequences *B *and *C *are independently homologous. Figure 8 illustrates the transitive homology between sequence *a *and sequence *b *through the third sequence c. The E-values between sequence *a *and sequence *c*, sequence *c *and sequence *b*, as well as sequence *a *and sequence *b *are 0.01, 0.005, 20 respectively. *P(a,c)*, *P(c,b)*, and *P(a,b) *are 0.8, 0.9, and 0.2 respectively. The homology between sequence *a *and sequence *b *cannot be detected with their direct E-value. However, the value of *P(a,b) *is assigned to 0.72 because of the transitive sequence homology.

### Hierarchical average linkage clustering

Once the metric value for every pair of protein sequences is calculated, the hierarchical average linkage clustering method is applied to cluster the protein sequences in the new metric space using the geometric mean as the merging rule.

Hierarchical average linkages uses the Unweighted Pair-Group Average (UPGA) [26] to measure the distance between clusters. Let *D*_*i*_, *i *= 1,2, ... *n*. denote the protein sequences contained in Cluster *D *and let *E*_*j*_, *j *= 1,2, ..., *m *denote the protein sequence contained in Cluster *E*. The geometric mean distance *G *between Cluster *D *and Cluster *E *is defined as Equation 4:

Equation 4:

G=∏i=1,j=1i=n,j=mP(Di,Ej)
 MathType@MTEF@5@5@+=feaafeart1ev1aaatCvAUfKttLearuWrP9MDH5MBPbIqV92AaeXatLxBI9gBaebbnrfifHhDYfgasaacH8akY=wiFfYdH8Gipec8Eeeu0xXdbba9frFj0=OqFfea0dXdd9vqai=hGuQ8kuc9pgc9s8qqaq=dirpe0xb9q8qiLsFr0=vr0=vr0dc8meaabaqaciaacaGaaeqabaqabeGadaaakeaacqWGhbWrcqGH9aqpdaqeWbqaaiabdcfaqjabcIcaOiabdseaeTGaeeyAaKMccqGGSaalcqWGfbqrliabdQgaQPGaeiykaKcaleaacqWGPbqAcqGH9aqpcqaIXaqmcqGGSaalcqWGQbGAcqGH9aqpcqaIXaqmaeaacqWGPbqAcqGH9aqpcqWGUbGBcqGGSaalcqWGQbGAcqGH9aqpcqWGTbqBa0Gaey4dIunaaaa@49A8@

The hierarchical average linkage clustering works in an iterative process: it begins with each protein sequence as a singleton cluster; during each iteration, it finds two clusters with the lowest metric value, then joins these two clusters into a new cluster, and updates the metric value between this new cluster and all others. This process iterates until the current lowest metric value exceeds the threshold.

## Authors' contributions

QM carried out the design and implementation of the method and wrote the manuscript. JDS compared the performances between CLUGEN and MCL. GWC and RC participated in the project. NRN directed and participated in the project and prepared the figures in the manuscript. All authors involved in reviewing and revising the manuscript and approved the final manuscript.
